# Carbamazepine-induced reversible vitiligo

**DOI:** 10.1016/j.jdcr.2022.05.042

**Published:** 2022-06-09

**Authors:** Luai M. Assaedi, Hussein M. Alshamrani, Renad A. Abbas, Fadi Ali Alghamdi

**Affiliations:** a Department of Dermatology, King Fahd Armed Forces Hospital, Jeddah, Saudi Arabia; b Department of Dermatology, King Abdulaziz University, Jeddah, Saudi Arabia

**Keywords:** carbamazepine, carbamazepine-induced vitiligo, drug-induced vitiligo, vitiligo, NB-UVB, narrowband ultraviolet B

## Introduction

Vitiligo, a dermatological disease that is psychologically debilitating, affects approximately 0.5% to 2% of the general population worldwide, with no predominance of sex or ethnicity.^1^ In Saudi Arabia, specifically in the western region, the prevalence of vitiligo is 1.5%.^2^ The exact cause of this condition is unknown. However, many hypotheses have been proposed to explain its pathogenesis. These hypotheses include both autoimmune and neural mechanisms. Vitiligo is mainly described as the selective loss of melanocytes, which results in milky-white macules.^1^ Drug-induced vitiligo is a rare side effect of multiple drugs from different pharmacological groups, including anticonvulsant drugs, such as carbamazepine.^3^ There are reported cases which show that carbamazepine is one of the medications that may trigger vitiligo.^3^ Carbamazepine works on the nervous system to control seizures, pain, and bipolar disorder. The commonly reported side effects of carbamazepine comprise nystagmus and blurred vision. Less common adverse events of carbamazepine include shuffling gait and unsteadiness.^4^ Vitiligo is one of the rare side effects of carbamazepine, which, to the best of our knowledge, has been reported in only 1 case report. Herein, we report an additional case of carbamazepine-associated vitiligo.

## Case report

**Fig 1:**
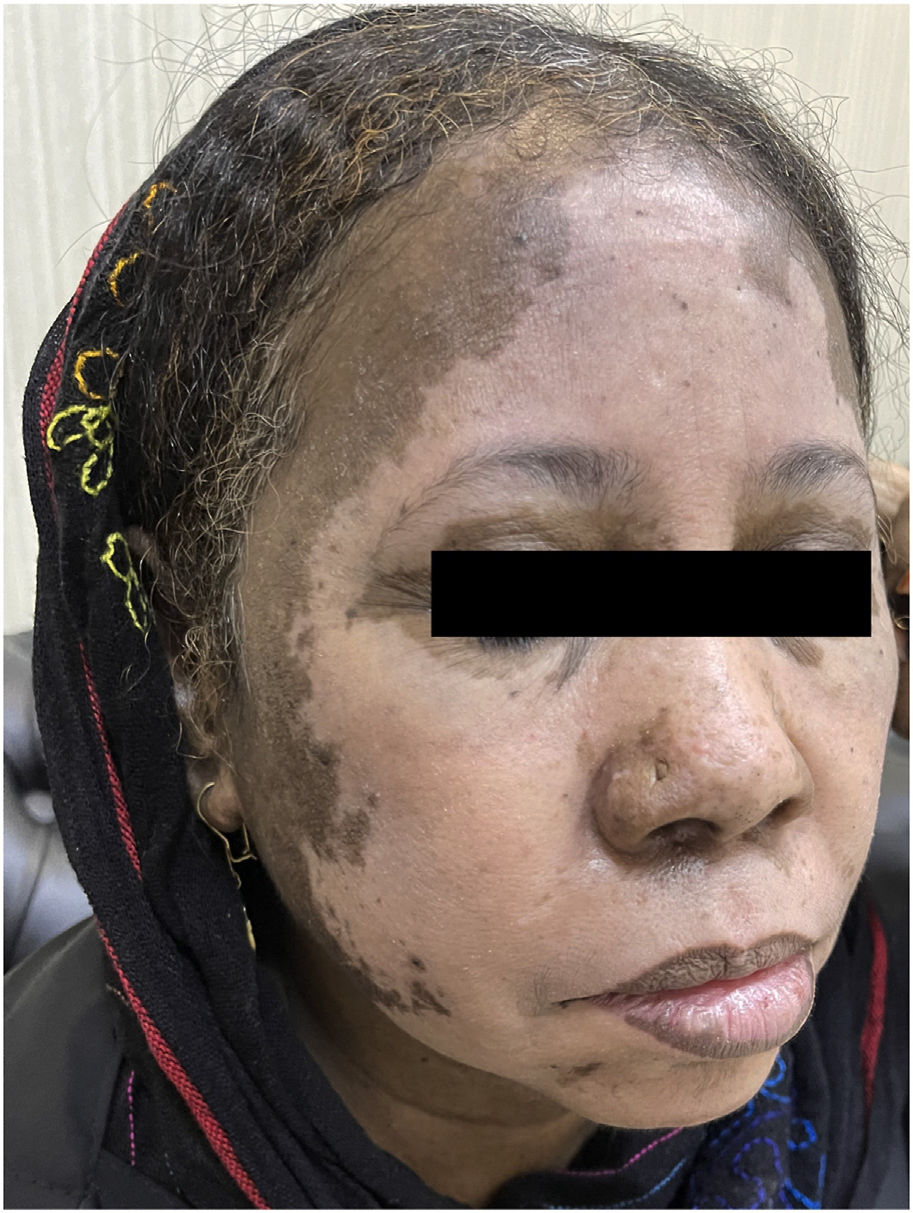
Carbamazepine-induced vitiligo. Depigmentation developed within 2 wk after carbamazepine administration.

A 60-year-old woman with a history of type 2 diabetes and hypertension who was on metformin and amlodipine had a cerebrovascular accident, which resulted in trigeminal neuralgia 1 mo later. She was prescribed carbamazepine (400 mg twice a day), gabapentin (300 mg once a day), and vitamin B complex tablets. She denied any topical or nonprescription treatment for her trigeminal neuralgia. She developed erythema over the face and redness over the body (face, hands, and legs), which evolved into depigmentation within 2 wk after initiating medication (Fig 1). The patient had no family history of vitiligo or thyroid disease.

When depigmentation occurred, carbamazepine was tapered down over 2 days. Moreover, the patient was administered duloxetine 60 mg once a day along with gabapentin and referred to the dermatology clinic for the appropriate management of depigmentation.

Physical examination revealed extensive symmetrical depigmented patches over the face and scattered small, ill-defined depigmented patches over the elbow and knee. Wood's lamp examination showed depigmented lesions that were accentuated with milky-white appearance. The patient was diagnosed with vitiligo, possibly due to carbamazepine. Therefore, she was started on narrowband ultraviolet B (NB-UVB). As an adjunct therapy, she was administered mometasone 0.1% cream 2 days a week for body lesions only and tacrolimus 0.1% ointment 5 days a week for both face and body lesions.

**Fig 2:**
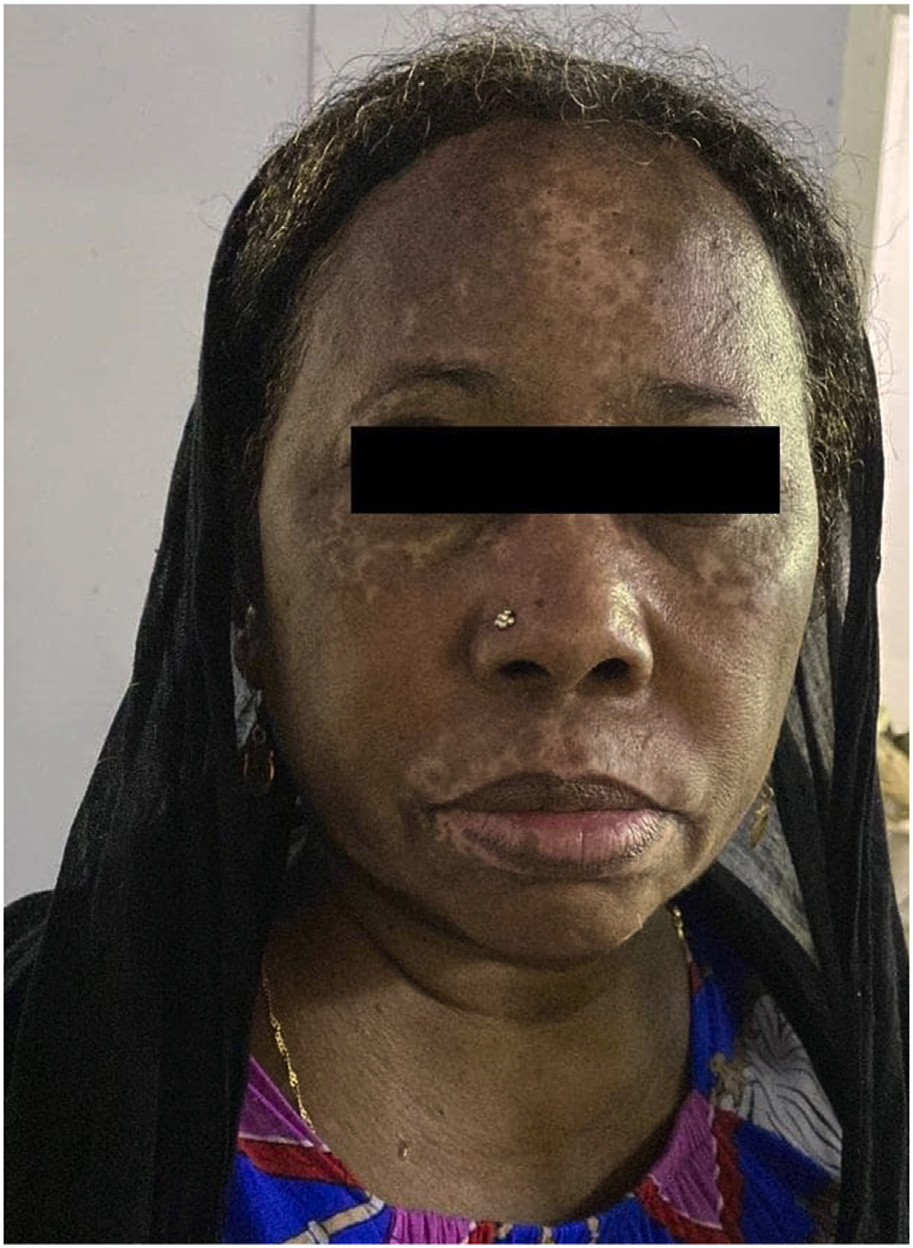
Carbamazepine-induced vitiligo. Skin repigmentation 2 mo following carbamazepine discontinuation.

The patient underwent 5 sessions of NB-UVB before ceasing treatment as she lived far from our hospital. The patient was re-evaluated 2 mo later; she showed almost complete repigmentation following carbamazepine discontinuation (Fig 2).

The patient declined rechallenge with carbamazepine.

## Discussion

Occurrence of vitiligo-like lesions is a reported side effect of multiple agents, including topical imiquimod, topical or intralesional corticosteroids,^5^^,^^6^ and systemic medications such as tyrosine kinase inhibitors.^7^ Other rare occurrences have been reported to be associated with administration of other therapeutic agents such as anticonvulsants and antimalarial medications.^3^

In this study, we report the case of a 60-year-old woman who developed depigmented lesions following the administration of carbamazepine for the treatment of trigeminal neuralgia.

The patient was diagnosed with drug-induced vitiligo, possibly due to carbamazepine use. Both reversibility of lesions and lack of use of any drugs other than her usual medications led to the suspicion that carbamazepine was the main culprit drug.

In contrast to classical vitiligo, drug-induced vitiligo in previous cases has been reported to have a predilection to sun-exposed areas.^8^ Our patient's main lesions were localized to sun-exposed skin, mainly over the face. However, she also developed symmetrical depigmented lesions on her elbows and legs. No progression or koebnerization was noted in the appearance of lesions.

Multiple hypotheses have been proposed regarding the pathogenesis of the disease in terms of melanocyte destruction and loss of function. These include neural, autoimmunity, genetics, oxidative stress, melanocyte self-destruction, and melanocyte detachment mechanisms.^1^ Many postulated theories for the pathogenesis of drug-induced vitiligo have been reported. They comprise activation of CD8+ (cytotoxic) T cells targeting melanocytes, direct cytotoxic and apoptotic effects of medications on melanocytes, and drug-induced damage and alteration to sympathetic nerves connected to melanocytes.^3^ Since most of the suggested pathogeneses of drug-induced vitiligo remain speculative, further studies are required to clarify its precise mechanism.

It is difficult to conclude the mechanism from a single case, as presented here. However, in our case, regaining of pigment upon carbamazepine discontinuation would seem to counter the theory of irreversible destruction of melanocytes or permanent impairment of sympathetic nerves. Since another case report described similar results of reversible vitiligo upon discontinuation of carbamazepine, this may be a unique adverse effect of this medication.^9^ Moreover, in the same previously reported case,^9^ the authors suggested that nonrecurrence of vitiligo with the reintroduction of carbamazepine may indicate immunity or desensitization to the drug. This observation may support theories against the direct cytotoxic effects of the drug on vitiligo induction. Further studies are needed to elucidate the exact mechanism underlying medication-induced vitiligo and draw a better conclusion regarding the mechanism by which some medications could impact melanocytes.

There is a limitation to our study. The patient received NB-UVB (for 5 sessions only) along with topical medications. Thus, this management could have helped prevent further progression of the disease.

In conclusion, we would like to emphasize the importance of paying attention to this side effect for establishing an early definitive diagnosis. In addition, prompt discontinuation of the causative medication could lead to resolution of depigmentation and prevent further damage that may affect the patient both physically and psychologically.

## Conflicts of interest

None disclosed.
